# Editorial: Evolutionary Mechanisms of Infectious Diseases

**DOI:** 10.3389/fmicb.2021.667561

**Published:** 2021-05-13

**Authors:** Jianying Gu, Zhan Zhou, Yufeng Wang

**Affiliations:** ^1^Department of Biology, College of Staten Island, City University of New York, New York, NY, United States; ^2^College of Pharmaceutical Sciences, Institute of Drug Metabolism and Pharmaceutical Analysis, Zhejiang University, Hangzhou, China; ^3^Department of Biology, South Texas Center for Emerging Infectious Diseases, University of Texas at San Antonio, San Antonio, TX, United States

**Keywords:** evolution, infectious diseases, infection, pathogenicity, virulence

Infectious diseases have been and continue to be one of the major causes of mortality around the world, posing significant health, social, and economic burdens. According to the World Health Organization (WHO), three infectious diseases (lower respiratory infections, diarrheal diseases, and tuberculosis) are among the top 10 causes of death. Infectious pathogens continue to emerge and re-emerge, underscoring considerable epidemic challenges to public health (Fauci, 2005; Watkins, 2018).

Evolution plays an important role in infectious diseases (Antia et al., 2003; Chabas et al., 2018; Echaubard et al., 2018). Driven by constant arms race between microbial pathogens and their hosts, pathogens evolve mechanisms to evade host defense (Hilleman, 2004; Van Avondt et al., 2015; Bernard et al., 2018), develop drug resistance (Nathan and Cars, 2014; Laxminarayan et al., 2016; Haldar et al., 2018), adapt to host environment (Sperandio, 2018; Beekman and Ene, 2020), compete with host microbiota (Vonaesch et al., 2018; Tsolis and Baumler, 2020), evolve virulence (Berngruber et al., 2013; Cressler et al., 2016; Geoghegan and Holmes, 2018), and spread and transmit to new hosts (McCallum et al., 2001; Antonovics et al., 2017). A better understanding of the key evolutionary features of infectious diseases such as pathogenicity, infectiousness, and transmissibility could result in effective prevention and control strategies.

Evolutionary processes of infectious diseases reflect temporal and spatial dynamic host-pathogen relationships, which may be revealed in genome structures and organization, population dynamics, and host/vector physiological systems. Recent efforts that bring together high-throughput omics technologies and big data analytical approaches have contributed to new insights of these processes (Cowell and Winzeler, 2019; Khan et al., 2019; Ball et al., 2020). The goal of this Research Topic is to provide a forum for sharing ideas, tools, and results among researchers from fields of evolutionary biology, infectious diseases, microbiology, genomics, and epidemiology. The Topic is mainly focused on recent progress in using multidisciplinary approaches to the studies of the evolutionary mechanisms of pathogenesis, virulence, host immunity, population dynamics, and epidemiology.

We sincerely thank all researchers who have contributed to our Research Topic. This collection of 21 articles is divided into four sections. The first section includes six articles focusing on Coronavirus disease 2019 (COVID-19) and its causative agent severe acute respiratory syndrome coronavirus 2 (SARS-CoV-2). As of February 2021, over 104 million COVID-19 cases have been confirmed worldwide, including over 2.2 million deaths (https://covid19.who.int/) since the emergence of the pandemic in December 2019 (Sohrabi et al., 2020; Yang et al., 2020). The six articles in this section offer new insight into the evolutionary and structural features of SARS-CoV-2: (1) Xing et al. develop a freely available program, MicroGMT, to identify and characterize mutations in microbial genomes, with the default setting optimized for SARS-CoV2. (2) Shen et al. present a comprehensive genomic epidemiology study that reveals a haplotype pattern of geographical specificity at the city-, state-, and country-levels, supporting the effectiveness of travel restriction in preventing widespread transmission. (3) Lv et al. describe a comparative genomic analysis between human SARS-CoV-2 and its close relative Bat-CoV RaTG13, and identify mutation patterns indicative of stronger purifying selection occurred in SARS-CoV-2. (4) The article by Liu S. et al. extends this topic. Distinct genomic single nucleotide variation signatures are identified in more than 30,000 SARS-CoV-2 genomes, which may have functional consequences due to the viral genetic instability (Liu S. et al.). (5) Readers interested in SARS-CoV-2 pathogenesis will appreciate a timely report by Liu T. et al. on the features and evolutionary difference of viral gene expression and fusion events in the SARS-CoV-2 infected cells from the patients with moderate and severe COVID-19. (6) Cui and Zhang report the presence of G-Quadruplexes in human coronaviruses including SARS-CoV-2, suggesting targeting G-Quadruplexes as a potential avenue to COVID-19 therapeutics.

The second section is devoted to the evolutionary mechanisms of bacteria. We are glad to present three review articles: Tuberculosis, one of the oldest known human infectious diseases, remains one of the major causes of mortality globally. Allué-Guardia et al. present a timely review to address the interactions of *Mycobacterium tuberculosis* with the lung environment and how these interactions may drive phenotypes of *M. tuberculosis* with a particular emphasis on drug resistant *M. tuberculosis*. Chomkatekaew et al. present the evidences and their perspective on the genetic and genomic diversity of *Burkholderia pseudomallei*, the cause of a lethal tropical disease melioidosis, and shed light on the evolutionary arms race between the bacterial pathogen and the host. The article by Tang et al. provides a succinct review regarding the pathogenesis and epidemiology of *Klebsiella pneumoniae*, the causative agent of a range of respiratory tract, urinary tract, and bloodstream infections. Two of the four original research articles are focused on evolutionary mechanisms of antibiotic resistance. The article by Black et al. highlights the increasing of non-carbapenemase producing Enterobacterales (NCPE) in south Texas. Their findings may have a direct impact on treatment regimens for patients. Javed et al. report a nice experimental evolution study of an extensively drug resistant (XDR) strain of *Pseudomonas aeruginosa*, an opportunistic pathogen leading to widespread infection and outbreak in hospitals. Their study demonstrates that the acquisition of antibiotic (colistin) resistance can affect the level of virulence (Javed et al.). Using a molecular evolutionary analysis approach, Yamaguchi et al. report the role of BgaA as a pneumococcal virulence factor in *Streptococcus pneumoniae*, a common cause of community-acquired pneumonia. Hassan et al. study and compare the genetic changes and adaptation that occur to two different *Burkholderia* species during co-infection of a cystic fibrosis patient's lungs over 4.4 years.

The third section is composed by seven articles on eukaryotic pathogens. Four articles are focused on malaria, a life-threatening mosquito-borne infectious disease. We highly recommend an excellent review article by Su et al. which addresses how the malaria parasites and their hosts interact, with a particular emphasis on the evolutionary impacts of recent human interventions such as drug and vaccine development. Three of the original articles address different important issues in malaria research: Brashear et al. present the draft of *de novo* genome assembly of four new *Plasmodium vivax* clinical samples collected in the China-Myanmar border area. The study provides new insight into genetic diversity and copy number variations of *P. vivax* multigene families (Brashear et al.). Huang et al. genotype the parasites from Southern China and present evidence for a genomic population with drug resistance. The study also demonstrates the utility of SNP microarrays for large-scale parasite molecular epidemiology (Huang et al.). Further on the application side, Mu et al. develop a novel, highly sensitive and accurate detection method for malaria parasite infection *via* integration of inductively coupled plasma mass spectrometry (ICP-MS) and Gold nanoparticles (AuNPs). Three articles in this section study evolutionary mechanisms of other eukaryotic pathogens. *Cryptosporidium* and *Giardia* cause intestinal illnesses in humans and animals. Wu et al. describe the first molecular epidemiological investigation of *Cryptosporidium* spp. and *G. duodenalis*, in humans in Myanmar, indicating the large potential of zoonotic transmission. The article by Palevich et al. describes a comprehensive analysis of the complete mitochondrial genomes of the New Zealand field strains of *Haemonchus contortus* and *Teladorsagia circumcincta*, two gastrointestinal nematodes. Using multilocus sequence typing, Liu X. et al. identify genetic diversity and clonal population structure in isolates of *Enterocytozoon bieneusi*, a unicellular microsporidian parasite closely related to fungi.

The last section of the collection includes an interesting piece of work by Shen-Gunther et al. on host-pathogen interactions. The article reports a large-scale validation study for the development of a panel of markers for the prediction of severity of cervical cancer (Shen-Gunther et al.). This panel, namely molecular pap smear, may have clinical applications since it captures pathogen and host specific signature related to pathogenicity and host susceptibility.

In summary, this collection of 21 articles covers a variety of topics in evolutionary mechanisms of infectious diseases, including the role of factors that influence pathogen virulence and host susceptibility, the role of genetic variation and population dynamics on pathogenesis, the role of medical interventions on drug resistance, and the role of disease control interventions on pathogen emergence and transmission. We hope that the Research Topic will be useful for a wide audience, particularly evolutionary biologists, microbiologists, infectious disease researchers and clinicians, genome scientists, systems biologists, graduate, and undergraduate students.

## Author Contributions

All authors listed have made a substantial, direct and intellectual contribution to the work, and approved it for publication.

## Conflict of Interest

The authors declare that the research was conducted in the absence of any commercial or financial relationships that could be construed as a potential conflict of interest.

## References

[B1] Antia R. Regoes R. R. Koella J. C. Bergstrom C. T. (2003). The role of evolution in the emergence of infectious diseases. Nature 426, 658–661. 10.1038/nature02104 14668863 PMC7095141

[B2] Antonovics J. Wilson A. J. Forbes M. R. Hauffe H. C. Kallio E. R. Leggett H. C.. (2017). The evolution of transmission mode. Philos. Trans. R. Soc. Lond. B. Biol. Sci. 372:20160083. 10.1098/rstb.2016.0083 28289251 PMC5352810

[B3] Ball B. Langille M. Geddes-McAlister J. (2020). Fun(gi)omics: advanced and diverse technologies to explore emerging fungal pathogens and define mechanisms of antifungal resistance. mBio 11. 10.1128/mBio.01020-20 33024032 PMC7542357

[B4] Beekman C. N. Ene I. V. (2020). Short-term evolution strategies for host adaptation and drug escape in human fungal pathogens. PLoS Pathog. 16:e1008519. 10.1371/journal.ppat.1008519 32407384 PMC7224449

[B5] Bernard Q. Smith A. A. Yang X. Koci J. Foor S. D. Cramer S. D.. (2018). Plasticity in early immune evasion strategies of a bacterial pathogen. Proc. Natl. Acad. Sci. U.S.A. 115, E3788–E3797. 10.1073/pnas.1718595115 29610317 PMC5910839

[B6] Berngruber T. W. Froissart R. Choisy M. Gandon S. (2013). Evolution of virulence in emerging epidemics. PLoS Pathog. 9:e1003209. 10.1371/journal.ppat.1003209 23516359 PMC3597519

[B7] Chabas H. Lion S. Nicot A. Meaden S. van Houte S. Moineau S.. (2018). Evolutionary emergence of infectious diseases in heterogeneous host populations. PLoS Biol. 16:e2006738. 10.1371/journal.pbio.2006738 30248089 PMC6171948

[B8] Cowell A. N. Winzeler E. A. (2019). Advances in omics-based methods to identify novel targets for malaria and other parasitic protozoan infections. Genome Med. 11:63. 10.1186/s13073-019-0673-3 31640748 PMC6805675

[B9] Cressler C. E. McLeod D. V. Rozins C. Van Den Hoogen J. Day T. (2016). The adaptive evolution of virulence: a review of theoretical predictions and empirical tests. Parasitology 143, 915–930. 10.1017/S003118201500092X 26302775 PMC4873896

[B10] Echaubard P. Rudge J. W. Lefevre T. (2018). Evolutionary perspectives on human infectious diseases: challenges, advances, and promises. Evol. Appl. 11,383–393. 10.1111/eva.12586 29636793 PMC5891049

[B11] Fauci A. S. (2005). Emerging and reemerging infectious diseases: the perpetual challenge. Acad. Med. 80, 1079–1085. 10.1097/00001888-200512000-00002 16306276

[B12] Geoghegan J. L. Holmes E. C. (2018). The phylogenomics of evolving virus virulence. Nat. Rev. Genet. 19, 756–769. 10.1038/s41576-018-0055-5 30305704 PMC7096893

[B13] Haldar K. Bhattacharjee S. Safeukui I. (2018). Drug resistance in plasmodium. Nat. Rev. Microbiol. 16, 156–170. 10.1038/nrmicro.2017.161 29355852 PMC6371404

[B14] Hilleman M. R. (2004). Strategies and mechanisms for host and pathogen survival in acute and persistent viral infections. Proc. Natl. Acad. Sci. U.S.A. 101(Suppl. 2), 14560–14566. 10.1073/pnas.0404758101 15297608 PMC521982

[B15] Khan M. M. Ernst O. Manes N. P. Oyler B. L. Fraser I. D. C. Goodlett D. R.. (2019). Multi-omics strategies uncover host-pathogen interactions. ACS Infect. Dis. 5, 493–505. 10.1021/acsinfecdis.9b00080 30857388 PMC13488112

[B16] Laxminarayan R. Sridhar D. Blaser M. Wang M. Woolhouse M. (2016). Achieving global targets for antimicrobial resistance. Science 353, 874–875. 10.1126/science.aaf9286 27540009

[B17] McCallum H. Barlow N. Hone J. (2001). How should pathogen transmission be modelled? Trends Ecol. Evol. 16, 295–300. 10.1016/S0169-5347(01)02144-9 11369107

[B18] Nathan C. Cars O. (2014). Antibiotic resistance–problems, progress, and prospects. N. Engl. J. Med. 371, 1761–1763. 10.1056/NEJMp1408040 25271470

[B19] Sohrabi C. Alsafi Z. O'Neill N. Khan M. Kerwan A. Al-Jabir A.. (2020). World Health Organization declares global emergency: a review of the 2019 novel coronavirus (COVID-19). Int. J. Surg. 76, 71–76. 10.1016/j.ijsu.2020.02.034 32112977 PMC7105032

[B20] Sperandio V. (2018). Pathogens' adaptation to the human host. Proc. Natl. Acad. Sci. U.S.A. 115, 9342–9343. 10.1073/pnas.1813379115 30190426 PMC6156631

[B21] Tsolis R. M. Baumler A. J. (2020). Gastrointestinal host-pathogen interaction in the age of microbiome research. Curr. Opin. Microbiol. 53, 78–89. 10.1016/j.mib.2020.03.002 32344325

[B22] Van Avondt K. van Sorge N. M. Meyaard L. (2015). Bacterial immune evasion through manipulation of host inhibitory immune signaling. PLoS Pathog. 11:e1004644. 10.1371/journal.ppat.1004644 25742647 PMC4351076

[B23] Vonaesch P. Anderson M. Sansonetti P. J. (2018). Pathogens, microbiome and the host: emergence of the ecological Koch's postulates. FEMS Microbiol. Rev. 42, 273–292. 10.1093/femsre/fuy003 29325027

[B24] Watkins K. (2018). Emerging infectious diseases: a review. Curr. Emerg. Hosp. Med. Rep. 6, 86–93. 10.1007/s40138-018-0162-9 32226656 PMC7100414

[B25] Yang X. Yu Y. Xu J. Shu H. Xia J. Liu H.. (2020). Clinical course and outcomes of critically ill patients with SARS-CoV-2 pneumonia in Wuhan, China: a single-centered, retrospective, observational study. Lancet Respir. Med. 8, 475–481. 10.1016/S2213-2600(20)30079-5 32105632 PMC7102538

